# Protein S-nitrosylation in the heart: Mechanisms, physiological roles, and pathological remodeling

**DOI:** 10.1016/j.redox.2026.104414

**Published:** 2026-09-23

**Authors:** Maxime Gouin-Gravezat, Doria Boulghobra, Antoine Grandperrin, Beatrice Alpha-Bazin, Olivier Cazorla, Cyril Reboul

**Affiliations:** a Avignon Université, LaPEC, UPR4278, Avignon, F-84000, France; b Université Paris-Saclay, CEA, INRAE, Département Médicaments et Technologies pour la Santé (DMTS), SPI, Bagnols-sur-Cèze, 30200, France; c PhyMedExp, Univ Montpellier, INSERM, CNRS, CHU de Montpellier, Montpellier, France

**Keywords:** S-nitrosylation, Nitric oxide, Excitation contraction coupling, Heart failure

## Abstract

Protein S-nitrosylation (SNO), a reversible nitric oxide (NO)-dependent post-translational modification to reactive cysteine residues, finely tunes cardiac excitation–contraction coupling, mitochondrial function, and stress responses. In cardiomyocytes, compartmentalized NO signaling via neuronal (nNOS), endothelial (eNOS), and inducible (iNOS) isoforms regulates SNO of key proteins (e.g., RyR2, LTCC, SERCA2a, mitochondrial Complex I). Under physiological conditions, SNO modulates Ca^2+^ handling, myofilament sensitivity, and bioenergetics, while pathological SNO dysregulation, hypo-nitrosylation in heart failure (HF) with reduced ejection fraction or hypernitrosylation in HF with preserved ejection fraction, contributes to contractile dysfunction, arrhythmias, and mitochondrial impairment. Cardioprotective SNO (e.g., during ischemia-reperfusion) acts as a “redox shield”, preventing irreversible oxidation of critical cysteines, whereas excessive or mislocalized SNO can be detrimental, this dynamic modification plays an essential role in maintaining cardiac function. However, disruption of NO bioavailability and redox balance during cardiovascular disease alters the cardiac S-nitrosoproteome, contributing to maladaptive signaling, mitochondrial dysfunction, contractile impairment, and pathological remodeling. Increasing evidence also highlights the dual nature of SNO exerts either protective or detrimental effects depending on the molecular context, subcellular localization, and disease stage. This review summarizes current knowledge on SNO mechanisms in the heart and discusses its roles in physiological cardiac function and major cardiac pathologies.

## Highlights

• Recent advances in S-nitrosoproteomics are reshaping our understanding of cardiac S-nitrosylation.
• NOS isoform localization determines compartment-specific S-nitrosylation signaling.
• Cardiac protein S-nitrosylation is tightly regulated in a target- and redox-dependent manner.
• Protein S-nitrosylation orchestrates the cardiac response to physiological and pathological stress.
• Disrupted NO homeostasis drives aberrant S-nitrosylation and cardiac remodeling.

## List of abbreviations

**ANT1**: adenine nucleotide translocase 1
**Ca^2+^**: calcium
**CaM**: calmodulin
**CaMKII**: Ca^2+^/calmodulin-dependent protein kinase II
**Casp-3**: caspase-3
**Cav-3**: caveolin-3
**cGMP**: cyclic guanosine monophosphate
**CICR**: Ca^2+^-induced Ca^2+^ release
**Cx43**: connexin 43
**CysNO**: S-nitrosocysteine
**CysTMT**: cysteine-reactive tandem mass tags
**CypD**: cyclophilin D
**DRP1**: dynamin-related protein 1
**ECC**: excitation–contraction coupling
**eNOS**: endothelial nitric oxide synthase
**ESI**: electrospray ionization
**ERβ**: estrogen receptor-β
**FAO**: fatty acid oxidation
**GSNO**: S-nitrosoglutathione
**GSNOR**: S-nitrosoglutathione reductase
**GRK2**: G-protein-coupled receptor kinase 2
**HF**: heart failure
**HFD**: high-fat diet
**HFpEF**: heart failure with preserved ejection fraction
**HFrEF**: heart failure with reduced ejection fraction
**HIF-1α**: hypoxia-inducible factor-1α
**I/R**: ischemia/reperfusion
**ICa,L**: L-type calcium current
**ICAT**: isotope-coded affinity tags
**iNOS**: inducible nitric oxide synthase
**IP_3_**: inositol 1,4,5-trisphosphate
**iTRAQ**: isobaric tags for relative and absolute quantitation
**l****-NAME**: Nω-nitro-l-arginine methyl ester
**L-VNIO**: vinyl-L-N-5-(1-imino-3-butenyl)-l-ornithine
**LC-MS/MS**: liquid chromatography–tandem mass spectrometry
**LTCC**: L-type Ca^2+^ channel
**MCUc**: mitochondrial Ca^2+^ uniporter complex
**MS**: mass spectrometry
**MS/MS**: tandem mass spectrometry
**mPTP**: mitochondrial permeability transition pore
**nNOS**: neuronal nitric oxide synthase
**Nav1.5**: voltage-gated sodium channel Nav1.5
**NCX**: sarcolemmal sodium–calcium exchanger
**NO**: nitric oxide
**NOS**: nitric oxide synthase
**PKA**: protein kinase A
**PKC**: protein kinase C
**PKG**: cGMP-dependent protein kinase
**PLB**: phospholamban
**PTMs**: post-translational modifications
**ROS**: reactive oxygen species
**RyR2**: ryanodine receptor
**SERCA2a**: sarcoplasmic reticulum Ca^2+^-ATPase 2a
**SNO**: S-nitrosylation
**sGC**: soluble guanylate cyclase
**SR**: sarcoplasmic reticulum
**TCA**: tricarboxylic acid cycle
**TMT**: tandem mass tag
**tPA**: tissue plasminogen activator
**TnC**: troponin C
**TnI**: troponin I
**Trx**: thioredoxin
**β-AdR**: β-adrenergic receptor
**β_3_-AdR**: β_3_-adrenergic receptor
**VSMCs**: vascular smooth muscle cells

## 1 Introduction

The heart is a highly specialized contractile organ that adapts its function to meet the metabolic demands of the body. Its mechanical activity relies on cardiomyocytes, where membrane depolarization triggers calcium (Ca^2+^) influx through L-type Ca^2+^ channels (LTCC). This Ca^2+^ influx acts as a signal to induce a larger release from the sarcoplasmic reticulum (SR) via ryanodine receptors (RyR), a process known as Ca^2+^-induced Ca^2+^ release. The resulting Ca^2+^ transient regulates sarcomere contraction after Ca^2+^ binds to troponin C (TnC), displacing troponin I (TnI) and allowing actin-myosin interaction, and stimulates mitochondrial ATP production by activating key dehydrogenases of the Krebs cycle. Relaxation begins when Ca^2+^ concentration decreases in the cytosol, causing Ca^2+^ to dissociate from TnC, followed by its removal via the sarcoplasmic reticulum Ca^2+^-ATPase (SERCA2a) and the sarcolemmal sodium-calcium exchanger (NCX). This tightly regulated cycle of contraction (systole) and relaxation (diastole), the excitation–contraction coupling (ECC), is modulated by post-translational modifications (PTMs) that fine-tune the activity of key proteins.

Among these PTMs, nitric oxide (NO) signaling has emerged as an important modulator of cardiomyocyte function. While NO's role in regulating vascular tone, recognized by the 1998 Nobel Prize, is well established, its functions within cardiomyocytes are less defined. Three isoforms of nitric oxide synthase (NOS) are expressed in cardiomyocytes, and recent studies have reported that membrane depolarization can trigger a NOS-dependent NO transient [1]. Historically, NO signaling in cardiomyocytes has been linked to the canonical soluble guanylate cyclase (sGC)-cyclic guanosine monophosphate (cGMP)-dependent protein kinase (PKG) pathway [2,3]. However, NO also directly modulates cellular function via S-nitrosylation (SNO) of proteins involved in ECC. The labile and reversible nature of this redox-based PTM makes its quantification technically challenging, contributing to ongoing debates about SNO's functional role in cardiomyocytes.

Here, we critically review how SNO of cardiomyocyte proteins regulates ECC and myocardial function, from physiological adaptations to pathological remodeling.

## 2 Methods to measure protein S-nitrosylation

The discovery of NO as a signaling molecule, recognized by the 1998 Nobel Prize, greatly stimulated research into protein SNO. However, the detection and characterization of SNO remain analytically challenging. The Cys–NO covalent bond is labile and reversible and can be destabilized by light, high temperature, redox-active metal ions, reducing agents, and nucleophiles [4,5]. In addition, endogenous SNO modifications are generally of low abundance, while accurate characterization often requires identification of the specific modified cysteine residue, particularly in proteins containing multiple potential SNO sites. Consequently, numerous analytical strategies have been developed over the past two decades to improve SNO detection, site identification, enrichment, and quantification (Fig. 1A).

**Fig. 1:**
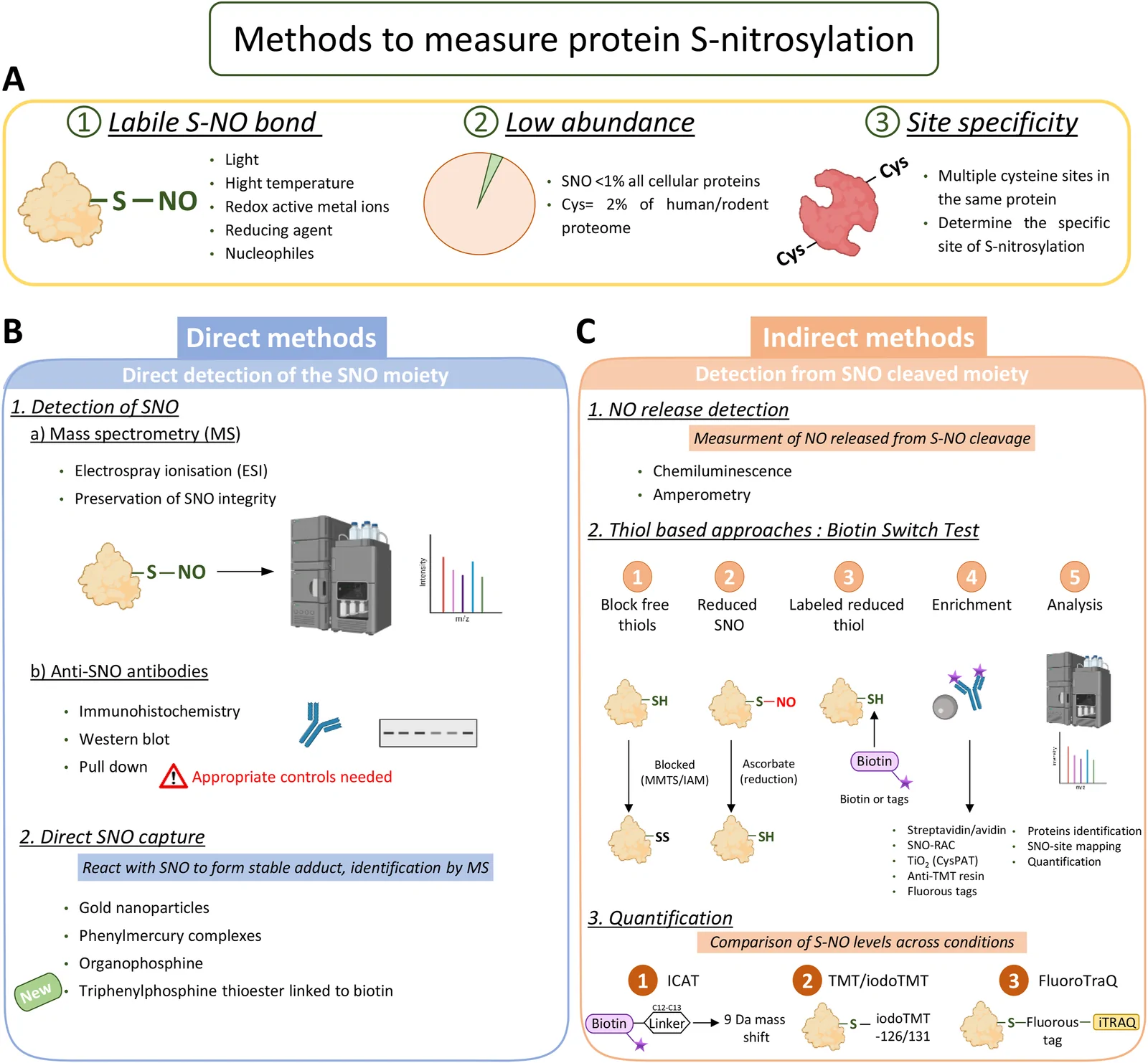
**S-Nitrosylation measure methods.** Due to the highly labile, low-abundance and redox-sensitive properties of S-nitrosylation, the reliable detection, quantification, and identification of SNO sites remain major analytical challenges. Direct methods enable the detection of SNO-modified proteins through approaches such as electrospray ionization mass spectrometry (ESI–MS) and anti-SNO antibodies. The formation of stable adducts through direct chemical reactions with SNO groups improves their stability and facilitates detection. Indirect methods rely either on the cleavage of the S–NO bond or on the replacement of the SNO moiety with a more readily detectable tag. In particular, the biotin-switch technique involves blocking free thiols, selectively reducing SNO groups, and replacing them with biotin, enabling enrichment and subsequent identification by mass spectrometry. Quantitative proteomic approaches further allow the comparison of SNO-modified peptide abundance across different biological conditions.

In direct strategies, the SNO entity is detected as a whole [6]. Mass spectrometry (MS) and the use of anti-SNO antibodies are straightforward methods (Fig. 1B). For MS analysis, electrospray ionization (ESI) conditions can be optimized to preserve the labile S–NO bond [7]. For anti-SNO antibodies, Western blotting and pull-down experiments require appropriate controls because of potential interference from other oxidative modifications [[8], [9], [10]]. Chemical approaches have also exploited the reactivity of SNO groups toward sulfur-affine reagents to generate stable adducts suitable for enrichment. Direct method strategies also introduce new chemical groups that react directly with SNO to form a stable adduct. Gold nanoparticles [11,12], phenylmercury complexes [[13], [14], [15]], and phosphine-based probes [[16], [17], [18], [19], [20]] have been used for this purpose, including biotinylated phosphine probes enabling affinity-based enrichment of SNO-modified proteins.

Indirect approaches, which detect products generated following cleavage of the S–NO bond, are more widely used (Fig. 1C). S-nitrosothiols can be quantified through the NO released after reduction, traditionally using chemiluminescence [21] and, more recently, by amperometry [22]. For free thiols, the gold standard technique is the biotin switch test (BST) for the detection and identification of SNO-modified proteins. This method relies on the specific labelling of thiols derived from SNO with a tag for their enrichment, detection, and the identification of SNO-bound proteins by MS [23]. BST involves three main steps: blocking free thiols, selectively reducing SNO groups to regenerate thiols, and labeling these newly formed thiols with a biotin-containing reagent. Biotinylated proteins can subsequently be enriched using avidin or streptavidin and analyzed by immunoblotting or MS. The captured biotinylated proteins can be efficiently eluted from the support by cleaving the disulfide bond with a reducing agent. Over time, the BST method has been refined. Improvements to BST-modified methods have focused on identifying SNO sites and overcoming limitations in sensitivity, specificity, and quantification. MS plays a key role in identifying SNO proteins and modification sites. Initially, MS analyses were performed at the protein level using MALDI-TOF MS and ESI-QTOF MS. These analyses have evolved alongside technological and instrumental progress in the field. Proteomics approaches such as tandem MS have enabled the identification of S-nitrosylated proteins within complex mixtures. These techniques involve the proteolytic digestion of proteins, the enrichment of S-nitrosylated peptides, and subsequent MS analysis. Hao et al. first described the modification of the BST for SNO-site identification, called BST/SNO-SID [24,25]. After BST, the biotinylated proteins are digested with trypsin. The resulting peptides are affinity-purified with immobilized monomeric avidin or streptavidin, eluted, and analyzed by LC–MS/MS. Elution from (strept)avidin agarose is performed with a reductant [24] or an acid that preserves bound biotin [10,26]. Interestingly, the enrichment step was more efficient when performed on tryptic peptide digests than on SNO-modified proteins [24]. To improve the accuracy of BST, the concentrations of the chemical reagents and the incubation time were adjusted to better complete the blocking step and accelerate the SNO reduction step [7,[27], [28], [29]]. Specificity and sensitivity of detection were improved by performing the reactions in darkness and introducing proper controls with the omission of the blocking reagent, the SNO-reducing reagent, and/or the labeling reagent [30]. Regarding the biotin labelling step, reversible disulfide bond formation in the BST protocol was replaced with irreversible S-alkylation to prevent interference from intermolecular disulfide bonds and improve specificity [31]. This irreversible covalent labelling method solved the issue of disulfide bond exchange and/or cleavage during MS identification. Strong reducing reagents, such as DTT and TCEP, can be used under these conditions to denature and digest proteins thoroughly, increasing sensitivity [32].

Quantification is required to compare SNO levels in different biological conditions. Quantitative MS methods are available, including label-free and stable-isotope labelling, which have been used in BST protocol variants [33]. To compare two conditions, BST is combined with isotope-coded affinity tags (ICAT). ICAT labels the cysteine thiols with an iodoacetamide group connected to biotin by a 12C (light ICAT) or 13C (heavy ICAT) linker containing 12 or 13 carbons, respectively, showing a mass difference of 9 Da. The biotin tag is acid-cleavable and provides higher-quality MS/MS spectra for identifying and quantifying SNO peptides [[34], [35], [36]]. In larger multiplex conditions, iodoacetyl tandem mass tag (TMT) reagents were used in a modified BST. IodoTMT consists of an iodoacetyl group, an MS-neutral spacer arm, and a tandem mass spectrometry (MS/MS) reporter with a mass ranging from 126 (iodoTMT-126) to 131 (iodoTMT-131) [37]. In these quantitative approaches, the differently labeled samples are pooled, trypsin-digested, and enriched using either streptavidin resin or anti-TMT antibody resin before liquid chromatography-tandem mass spectrometry (LC-MS/MS) analysis to identify peptides and quantify the relative abundance of reporter ions. The other commonly used reagents for multiplex quantitative analysis are Isobaric Tags for Relative and Absolute Quantitation (iTRAQ). Like TMT, these reagents consist of a reporter group, a balance group, and a reactive group that forms different isobaric mass tags. In 2020, the approach termed FluoroTraQ was reported. It combines a BST-modified approach, in which a fluorous tag switch technique is used to label SNO sites, a fluorous solid phase for enrichment, and iTRAQ labeling after trypsin digestion for multiplexed quantification [38]. In BST, biotinylated SNO proteins can be enriched via avidin/streptavidin agarose affinity capture. The proteins of interest are then detected by immunoblotting. Despite the popularity of the method, the modification sites are difficult to locate. Several BST variants have been developed to improve enrichment. SNO-RAC [38] uses thiol-reactive resins to capture reduced SH groups directly, bypassing the biotinylation steps. Trypsin digestion is performed on the resin to isolate peptides containing SNO sites. Thus, SNO-RAC offers a simplified approach for SNO detection. In addition to decreasing analysis time, SNO-RAC has demonstrated greater sensitivity than the BST method [39]. The CysPat method uses a cysteine-specific phosphonate adaptable tag to label nascent thiols, in combination with titanium dioxide chromatography enrichment, prior to nLC-MS/MS analysis [40]. Enrichment by TiO_2_ chromatography is highly efficient and specific [41,42]. It is also easy to use and inexpensive. Regarding cysteine-reactive tandem mass tags (Cys-TMT), the iodoTMT switch assay leads to the irreversible labeling of SNO-Cys sites, and the enrichment step is performed using high-affinity anti-TMT-bound agarose or chromatography and competitive elution [37]. The fluorous tag methodology is extremely selective, and its efficiency has been demonstrated in the enrichment of labeled peptides in proteomics [43]. The tag shows no fragmentation during MS/MS, and the reagents are relatively inexpensive. Beyond the quantification of changes in SNO levels, SNO occupancy, defined as the fraction of a specific cysteine residue within a given protein that is S-nitrosylated at a given time, is an important determinant of the functional impact of this post-translational modification [44]. Kohr et al. pioneered a quantitative proteomic approach to determine the absolute SNO occupancy of individual cysteine residues. Their method relied on CysTMT to differentially label free and SNO-modified thiols, before their quantification by MS [45]. Doulias et al. developed an alternative approach involving the direct reaction of phenyl mercury with SNO-modified cysteine residues and unblocked free thiol groups [46].

While experimental methods can be time-consuming and costly, computational approaches that aim to predict SNO sites have attracted growing interest and efforts. In this dynamic field, new approaches to algorithms are continuously implemented to more effectively respond to the challenge of predicting SNO sites. New tools build on the strengths and weaknesses of their predecessors while leveraging the latest technological advancements in the field. Several such tools have already been reported, including GPS-SNO and SNO-PseAAc, which are based on traditional machine learning algorithms [47,48], and DeepNitro and SNO-DCA, which use deep learning algorithms [49,50].

## 3 Mechanisms governing S-nitrosylation in cardiomyocytes

### 3.1 NOS expression and subcellular localization in cardiac myocytes

In the 1990s, all three NOS isoforms, constitutive neuronal (nNOS) and endothelial (eNOS), as well as inducible NOS (iNOS), were detected in biopsies from human hearts with dilated cardiomyopathy [51], and later in pathological and healthy rodent hearts [52,53].

In cardiomyocytes, the specific structural organization of the cell supports a highly compartmentalized distribution of NOS isoforms, enabling isoform-specific and spatially restricted NO signaling. This spatial confinement allows fine regulation of key cardiomyocyte functions, including energy metabolism, ECC, and response to stress [[54], [55], [56]]. Importantly, during adrenergic stress, eNOS and nNOS have been shown to exert independent –and in some cases opposing– effects on cardiac structure and function [57]. In addition, recent integrative analyses of NOSs interactomes and S-nitrosoproteomes revealed that NOS isoforms possess distinct interaction networks and that only a limited fraction of S-nitrosylated proteins directly interact with their NOS [58]. These findings underscore the importance of subcellular localization, rather than direct protein-NOS interaction, in shaping NO-SNO-dependent signaling.

The aim of this section is therefore to discuss how subcellular localization of NOS isoforms could contribute to regulate NO-SNO-dependent signaling in cardiomyocytes (Fig. 2, Fig. 3).

**Fig. 2:**
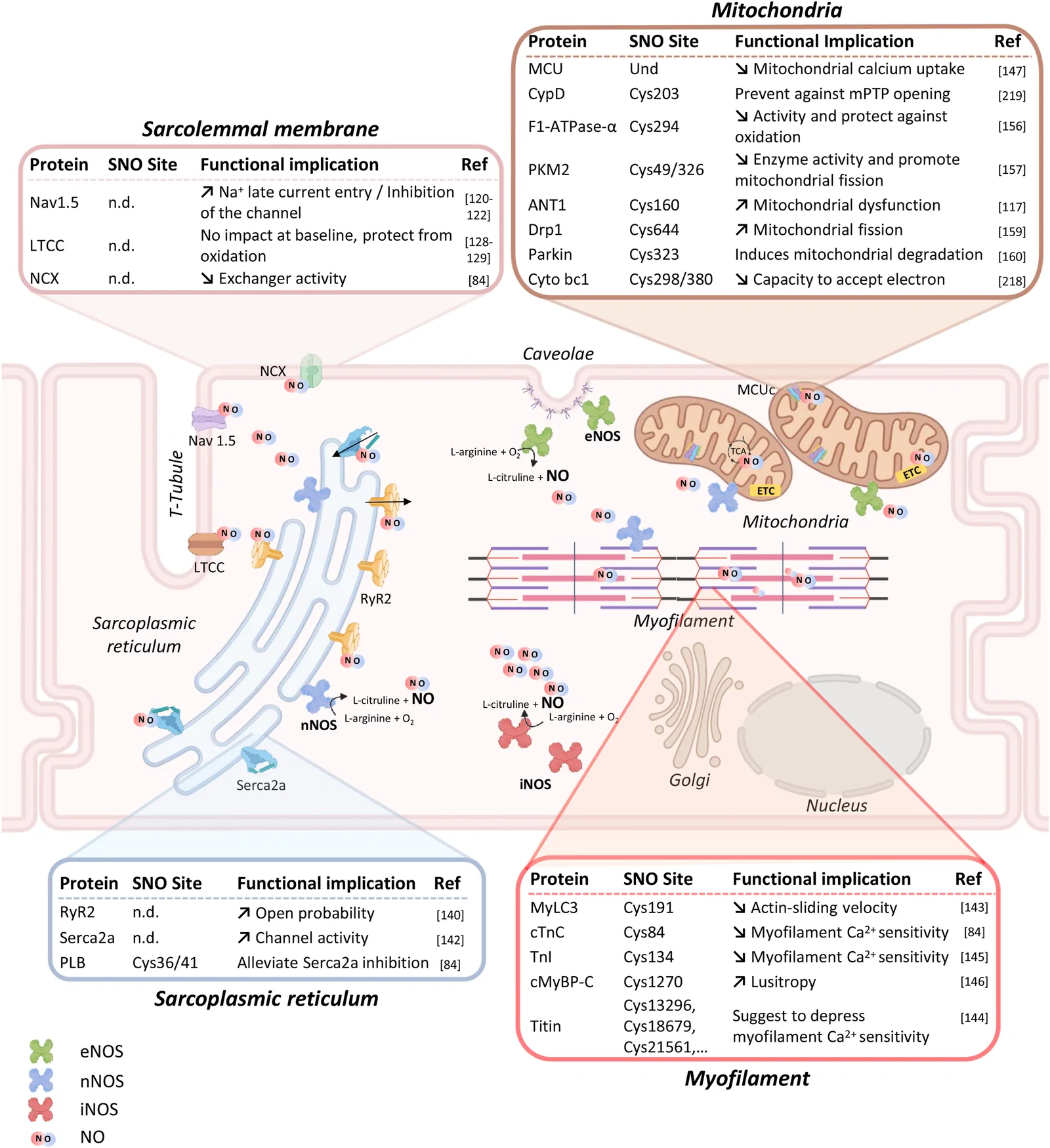
**Principal S-Nitrosylation sites within cardiomyocytes.** Overall, the effects of S-nitrosylation are highly dependent on its molecular targets and the specific cysteine residues modified. At the sarcoplasmic reticulum, S-nitrosylation appears to enhance Ca^2+^ handling by increasing both RyR open probability and SERCA activity. At the level of the myofilaments, S-nitrosylation seems to improve lusitropy by decreasing myofilament Ca^2+^ sensitivity. Within mitochondria, S-nitrosylation appears to modulate electron transport chain activity, regulate mitochondrial fission and mitophagy, and limit protein oxidation. Finally, at the plasma membrane, the effects of S-nitrosylation on ion transport remain poorly understood.

**Fig. 3:**
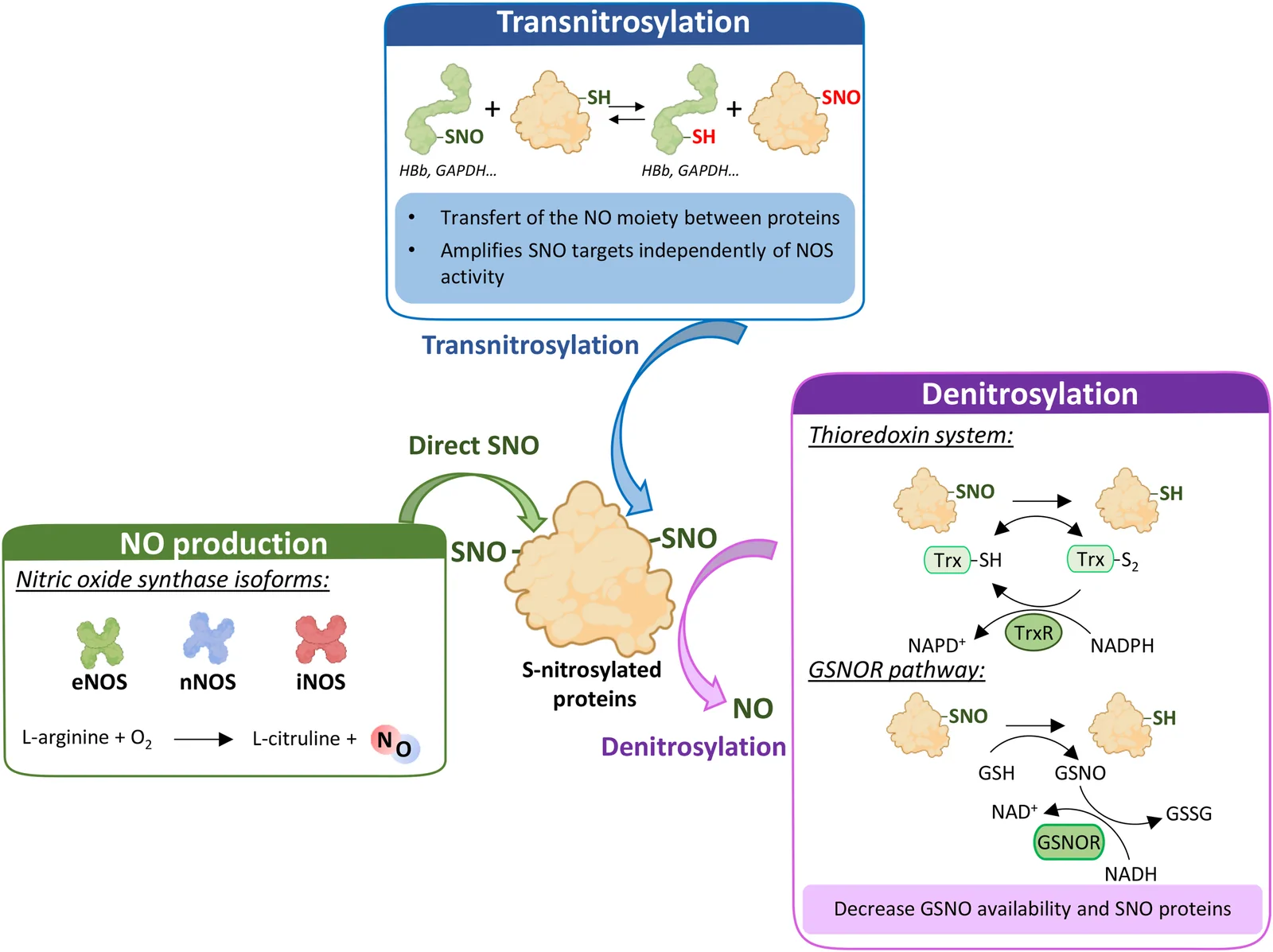
**S-nitrosylation homeostasis signaling.** S-nitrosylation (SNO) of cysteine residues is a tightly regulated process that governs its formation (S-nitrosylation), transfer (transnitrosylation), and removal (denitrosylation). The different nitric oxide synthase (NOS) isoforms—endothelial (eNOS), neuronal (nNOS), and inducible (iNOS)—mediate the production of nitric oxide (NO) from l-arginine at distinct concentrations and subcellular localizations. Transnitrosylation is a mechanism that transfers an NO moiety from one protein to another through intermediates such as hemoglobin (Hb) or glyceraldehyde-3-phosphate dehydrogenase (GAPDH). Independently of NOS activity, these mechanisms increase the pool of S-nitrosylated proteins. Denitrosylation is a finely regulated process mediated by thioredoxin (Trx) and S-nitrosoglutathione reductase (GSNOR), which remove NO groups from cysteine residues through redox reactions.

#### 3.1.1 Subcellular localization of eNOS in cardiomyocytes

The eNOS activity is tightly regulated by multiple PTMs, including phosphorylation, SNO, S-glutathionylation, and acetylation [56,59], which collectively modulate its enzymatic activity, protein–protein interactions, and subcellular localization. Beyond PTMs, subcellular compartmentalization is a key determinant of eNOS signaling [53,56,60], although it remains poorly explored in cardiomyocytes.

In cardiomyocytes, eNOS localizes primarily to sarcolemma caveolae, where caveolin-3 (Cav-3) inhibits its activity [61]. Ca^2+^-calmodulin (CaM) binding relieves this inhibition, restoring eNOS activity [62]. Caveolar integrity is essential for eNOS activation and cardioprotection, as its disruption impairs eNOS signaling and abolishes ischemic preconditioning-induced protection [63]. Consistently, β_3_-adrenergic receptors (β_3_-AdR) co-localize with Cav-3 and eNOS in caveolae [64] and mediate negative inotropic effects via the eNOS/NO/sGC/PKG pathway [65], an effect lost in β_3_-AR^−/−^ [66] or eNOS^−/−^ hearts [57].

Myristoylation and palmitoylation anchor eNOS to the membrane and caveolae [[67], [68], [69]], regulating its activation kinetics and kinase accessibility [70]. These lipid modifications also control eNOS trafficking to the Golgi [71], nucleus [60], and mitochondria [[72], [73], [74]]. At these sites, eNOS contributes to vesicular trafficking via N-ethylmaleimide-sensitive factor SNO [75], nuclear signaling [76], and mitochondrial function, with translocation occurring notably during exercise or ischemic preconditioning in a Cav-3–dependent manner [63]. In cardiomyocytes, β_3_-AdR stimulation also induces nuclear NO production [77].

Additional mechanisms further restrain eNOS activity through spatial sequestration. The HSP90/CHIP axis redirects eNOS toward inactive compartments [78], while NOSIP and NOSTRIN promote its cytoskeletal sequestration [62].

Overall, eNOS is a highly dynamic enzyme whose function depends on the interplay between PTMs and precise subcellular localization. However, most of these mechanisms have been characterized in endothelial cells and remain to be confirmed in cardiomyocytes.

#### 3.1.2 Subcellular localization of nNOS in cardiomyocytes

In cardiomyocytes, nNOS is better characterized than eNOS. Although both enzymes are constitutive and CaM-dependent, their regulation differs markedly. nNOS activity is tightly controlled by phosphorylation: Ser1412 enhances activation, while Ser847/Ser852/Ser741 phosphorylation inhibits activity, an effect reversed by protein phosphatase 1 [79,80].

Under physiological conditions, nNOS primarily localizes to the SR, where it regulates ECC [57,81]. At the SR, nNOS colocalizes with SERCA and enhances cardiac relaxation by relieving phospholamban (PLB)-mediated inhibition through phosphorylation at Ser16/Thr17 [82] and SNO at Cys36/Cys41 [83,84]. Additionally, nNOS interacts with the ryanodine receptor 2 (RyR2), modulating its function through SNO and phosphorylation [85].

nNOS signaling is further diversified by alternative splicing. The heart expresses four isoforms: nNOSα, β, μ, and ɣ [86,87]. Isoforms lacking exon 2 (nNOSβ, ɣ) lack a PDZ domain and are excluded from membrane and SR compartments [88], whereas PDZ-containing isoforms (nNOSα, μ) localize to these domains [55,86]. Functional studies indicate isoform-specific roles: nNOSβ notably localize to myofilaments [55], and isoform expression differentially impacts contractility [87].

Beyond the SR and sarcolemma, nNOS has also been identified in cardiac mitochondria, where it produces NO on a beat-to-beat basis and may thereby modulate oxidative phosphorylation [89], Moreover, nNOS overexpression increases mitochondrial nNOS content [83]. Although nuclear localization has not been demonstrated in cardiomyocytes, studies in skeletal muscle and neurons suggests potential nuclear roles, including regulation of CREB/PGC-1α signaling and transient nuclear translocation [90,91].

In pathology such as dilated cardiomyopathy and heart failure (HF), nNOS relocalizes from the SR to the sarcolemma via its N-terminal PDZ domain, interacting with membrane proteins [85]. This redistribution, coupled with reduced eNOS expression, promotes Cav-3 binding and disrupts Ca^2+^ handling and β-adrenergic signaling [92]. Functionally, sarcolemmal nNOS decreases LTCC activity and contributes to β-adrenergic hyporesponsiveness, which can be partially reversed by nNOS inhibition [85,93]. Additional interactions with sarcolemmal proteins like the calcium-calmodulin-dependent ATPase 4b pump and angiotensin receptors further modulate β-adrenergic responses, oxidative stress, and lusitropy [94,95]. Thus, PDZ-dependent relocalization of nNOS reshapes Ca^2+^ handling and cardiac signaling, potentially limiting excessive sympathetic stimulation in disease. In other pathology such as Duchenne muscular dystrophy in dogs (GRMD dogs), dystrophin deficiency disrupts the nNOS sarcolemmal localization, suggesting functional interactions between these proteins [96].

Collectively, these findings establish nNOS as a spatially and functionally compartmentalized enzyme, whose isoform diversity and dynamic localization finely tune cardiac signaling. They also support coordinated regulation between nNOS and eNOS within interconnected subcellular microdomains.

#### 3.1.3 Subcellular localization of iNOS in cardiomyocytes

iNOS, was first identified in macrophages as a key mediator of immune and inflammatory responses [97]. In cardiomyocytes, iNOS is induced by inflammatory stimuli such as myocardial infarction and HF [98,99]. Its expression is upregulated by pro-inflammatory cytokines (IL-1β, TNF-α, IFN-γ) or lipopolysaccharide via NF-κB activation [100], and is associated with inflammatory infiltration, fibrosis, and hypertrophy [101], underscoring its central role in pathological cardiac remodeling. However, its role in the heart remains debated, with some studies suggesting constitutive expression and potential involvement in basal cardiac function or ischemic preconditioning [54,102,103].

Structurally, iNOS binds CaM independently of Ca^2+^ with higher affinity than eNOS and nNOS, enabling activation at low Ca^2+^ concentrations and sustained NO production at micromolar levels [104]. This high NO output is a major source of nitro-oxidative stress, contributing to cardiac dysfunction [101].

The subcellular localization of iNOS remains poorly characterized in cardiomyocytes but appears to be regulated by PTMs. Although no direct evidence exists in cardiomyocytes, studies in skeletal muscle cells show that palmitoylation at Cys3 promotes membrane localization and activity, whereas its absence leads to Golgi or perinuclear accumulation and loss of function [105]. In normal cardiac tissue, iNOS has been detected predominantly in the mitochondria and contractile fibers, as well as along the plasma membrane and T-tubules [54]. Perinuclear sequestration, including aggresome formation, has also been reported and is associated with reduced responsiveness to inflammatory stimuli [106,107]. In addition, phosphorylation-dependent relocalization to insoluble cytosolic fractions further limits iNOS activity [108,109], highlighting the importance of PTMs in controlling its distribution and function. iNOS can also localize to mitochondria [110], where interaction with HSP22 promotes its translocation and enhances respiratory capacity [111]. Conversely, recent studies link iNOS to mitochondrial dysfunction in heart failure with preserved ejection fraction (HFpEF), transverse aortic constriction, and hypertension [99], suggesting context-dependent effects.

Overall, the role of iNOS in cardiomyocytes remains incompletely understood due to limited data. While most evidence supports a predominantly deleterious role in inflammatory cardiac remodeling, lower expression levels and specific subcellular localization may confer context-dependent regulatory or protective functions.

### 3.2 SNO, balance between transnitrosylation and denitrosylation

While NO production via NOS activity is considered a major driver of protein SNO, this redox PTM is also tightly regulated by transnitrosylation and denitrosylation (Fig. 3). Transnitrosylation (or trans-S-nitrosylation) is the enzymatic or chemical transfer of a NO group from an S-nitrosothiol (R-SNO) to a free thiol group (R′-SH), resulting in the formation of a new S-nitrosothiol (R′-SNO) and regeneration of the original thiol (R-SH). Hemoglobin, GAPDH, and caspase-3 (casp-3) have been previously proposed as transnitrosylating enzymes, transferring NO moieties between thiols [112]. Conversely, denitrosylation is catalyzed by enzymes such as S-nitrosoglutathione reductase (GSNOR), thioredoxin (Trx), and α-keto reductase family 1-member 1 [112]. This dynamic balance is essential for cardiovascular homeostasis.

GSNOR deficiency in mice produces multiple cardiovascular phenotypes: faster Ca^2+^ transient decay and accelerated sarcomeric relaxation [113], reduced peripheral vascular tone [114], enhanced neovascularization [113], and depressed β-adrenergic responses due to impaired β-agonist–induced denitrosylation of cardiac RyR2 [114]. Additionally, GSNOR knockout mice exhibit reduced myocardial infarct size, potentially linked to increased SNO of hypoxia-inducible factor-1α (HIF-1α) under normoxia and altered capillary density, implicating GSNOR in cardiac angiogenesis [115]. Interestingly, GSNOR inhibition or deletion is cardioprotective in male hearts but exerts opposite effects in females [116]. This sex specific difference is discussed below (8. Sex-dependent differences in cardiac S-nitrosylation).

Together, these findings strongly suggest that GSNOR critically modulates mitochondrial function under stress. Supporting this, GSNOR localizes to the mitochondrial matrix of cardiomyocytes [117], where it denitrosylates adenine nucleotide translocase 1 (ANT1) to preserve mitochondrial homeostasis in a mouse model of HF. Notably, GSNOR expression is suppressed in patients with HF [117]. More recently, a rat model of HFpEF (ZSF1 Ob) revealed nitrosative stress associated with increased cardiac hemoglobin subunit β (a transnitrosylating enzyme) expression, alongside reduced Trx2 and GSNOR activity [118].

Collectively, these findings demonstrate that transnitrosylation and denitrosylation enzymes like GSNOR and Trx regulate both cardiomyocyte excitation–contraction coupling and the global cardiac stress response. However, the precise interactions between NOS-derived NO signaling and protein transnitrosylation require further clarification.

## 4 S-nitrosylation and excitation-contraction coupling (Fig. 2)

### 4.1 S-nitrosylation and Ca^2+^ handling during the ECC

#### 4.1.1 SNO of sodium channels (Nav1.5)

Cardiac contraction begins with the membrane depolarization that activates the LTCC. Notably, the Nav1.5 α-subunit of the cardiac sodium channel, which initiates and propagates the action potential, coimmunoprecipitates with nNOS [119]. Under Cav-3 control, local nNOS-derived NO may S-nitrosylate Nav1.5, increasing Na^+^ influx [120]. However, NO donors inhibit voltage-gated Na^+^ channels in cardiomyocytes via SNO, as reducing agents (NEM or DTE) reverse this effect [121]. In pathology, high-level CO exposure, which induces fatal arrhythmias, is associated with NO-mediated SNO of Nav1.5 and increased late Na^+^ current [122]. In this model, Nω-propyl-l-arginine (l-NAME), a NOS inhibitor, normalized both peak and late Na^+^ currents, while DTT (which reverses SNO) abolished CO-induced arrhythmias in cardiomyocytes. Although Nav1.5 activity is regulated by SNO, the exact physiological relevance of this redox PTM requires deeper investigation.

#### 4.1.2 SNO of L-type Ca^2+^ channels

Following membrane depolarization, LTCCs open. In the 1990s, studies established that NO does not affect basal L-type calcium current (I_Ca,L_) but inhibits cAMP-stimulated I_Ca,L_, an effect attributed to the NO-cGMP-PKG pathway [123,124]. This paradigm was challenged when Campbell et al. [125] demonstrated SNO-dependent LTCC inhibition in ferret ventricular myocytes. Subsequent work in HEK cells expressing rabbit cardiac Ca^2+^ channels confirmed that the NO donor SNAP inhibits LTCC activity via a cGMP-independent, redox-sensitive mechanism involving direct SNO of cysteine residues, potentially explaining NO's negative inotropic effects [126]. In smooth muscle cells, NO-mediated SNO of L-type Cav1.2 Ca^2+^ channel reduces both Ca^2+^ current and channel surface expression via lysosomal degradation [127]. In ventricular cardiomyocytes, however, the role of SNO is less clear. While high NO concentrations from NO donors inhibit LTCC activity, nNOS inhibition with vinyl-L-N-5-(1-imino-3-butenyl)-l-ornithine (L-VNIO) increases intracellular Ca^2+^ transients without affecting LTCC activity [128]. Single-channel studies in artificial membranes confirm no direct NO effect on Cav1.2 [128], consistent with Vielma et al. [129] who found that acute β-adrenergic stimulation with isoproterenol increases total protein SNO without affecting Ca^2+^ channels. Yet, the same study showed that nNOS inhibition with SMTC or l-NAME decreases basal cardiac contractility and basal SNO of total proteins, including LTCC, suggesting constitutive SNO. Moreover, in a long-term isoproterenol stimulation model in tail-suspended rats, SNO of the LTCC α1C subunit competitively prevents reactive oxygen species (ROS)-mediated oxidation at the same sites, protecting the channel [130].

Thus, while the functional impact of LTCC SNO remains debated, with contradictory findings between NO donor applications and NOS inhibition, evidence suggests that SNO may protect channels from oxidative damage. Further research using more specific NO donors or conditional NOS isoform deletion is needed to clarify the physiological role of LTCC SNO.

#### 4.1.3 SNO of RyR2 channels

In cardiomyocytes, LTCC-mediated Ca^2+^ entry triggers SR Ca^2+^ release via RyR2 channels, a process known as Ca^2+^-induced Ca^2+^ release (CICR). CICR drives the rapid rise in cytosolic Ca^2+^ transient, which activates sarcomere contraction and stimulates mitochondrial ATP production. RyR2 open probability is a critical determinant of cardiac function. Increased open probability enhances the cytosolic Ca^2+^ transient during ECC, potentiating contraction in response to β-adrenergic stimulation. However, excessive open probability during diastole causes spontaneous RyR2 openings, leading to SR Ca^2+^ leak [131]. When such leaks become frequent, they are associated with delayed afterdepolarizations, a key trigger of cardiac arrhythmias [132] as well as mitochondrial Ca^2+^ overload [133], which contributes to mitochondrial ROS production [134] and subsequent RyR2 oxidation [135].

The impact of RyR2 SNO is complex and has been previously reviewed [136]. In vitro studies in lipid bilayers demonstrate that NO donor-mediated SNO of RyR2 such as S-nitrosoglutathione (GSNO) and S-nitrosocysteine (CysNO) increases channel open probability [137]. In cardiomyocytes, the effects of SNO on RyR2 depend on the localization of NOS isoforms. nNOS localizes to the SR and directly modulates RyR2 [57]. Studies in nNOS knockout mice (nNOS^−/−^) show decreased RyR2 SNO, increased oxidation, greater SR Ca^2+^ leak, reduced SR Ca^2+^ load, and a higher incidence of spontaneous Ca^2+^ waves, leading to ventricular arrhythmias [138]. Notably, this arrhythmogenic phenotype can be prevented by NO donors GSNO [138] or SNAP [139]. These findings highlight the complex redox regulation of RyR2, suggesting that physiological SNO maintains proper channel activity, whereas under oxidative stress, SNO may serve a protective role by preventing irreversible oxidation and RyR2 dysfunction. While eNOS is primarily localized in caveolae, its contribution to RyR2 regulation appears less clear. In a model of exercise-induced eNOS activation with no impact on nNOS, it is reported that the abundance of SNO-RyR2 is increased [140]. This suggests that the activation of eNOS in response to cardiac stress could also participate in the regulation of RyR2 through SNO.

#### 4.1.4 SNO of SERCA2a and NCX

Following contraction, Ca^2+^ is removed from the cytoplasm, mainly by SERCA2a and NCX, driving cardiac relaxation. The literature on SERCA2a SNO is contradictory and often methodologically flawed. For example, one study reported that myocardial relaxation is modulated by SERCA2a SNO [141], yet it relied on a polyclonal anti-SNO-Cys antibody, a less precise method than those described in “*Methods to measure protein S-nitrosylation”*. In addition, the study neither performed SERCA2a co-immunoprecipitation nor took precautions to preserve the highly labile SNO group. More rigorously, Sun et al. used the biotin-switch assay with proteomic analysis to show that cardiac ischemic preconditioning is associated with SERCA2a SNO [142]. In this work, GSNO-treated cardiomyocytes exhibited accelerated Ca^2+^ transient decay, consistent with enhanced SERCA2a activity. Consistently, PLB SNO, detected via biotin switch assay and resin enrichment and Western blotting, correlates with elevated SERCA2a activity under β-adrenergic stress, an effect abolished in GSNOR-overexpressing mice [84]. In the same model, NCX SNO was observed in response to isoproterenol but was absent in GSNOR-overexpressing mice. The authors also demonstrated that β-adrenergic stimulation upregulates NCX activity via protein kinase A (PKA)-induced phosphorylation, an effect limited by SNO in the wild-type hearts.

Overall, the literature on SNO and Ca^2+^ handling during the ECC suggests that multiple key proteins are prone to SNO, and this PTM modulates ECC (Fig. 2). SNO appears to temper Ca^2+^ handling during ECC, either directly by regulating Ca^2+^ entry and SR Ca^2+^ release, or indirectly by preventing protein oxidation and modulating kinase activity. However, interpreting the biological role of SNO signaling remains challenging when studies use untargeted NO donors, which flood cells with NO, contrasting with the highly compartmentalized nature of endogenous NO signaling [57]. To clarify SNO's role in cardiac myocytes, more specific approaches such as targeted NOS inhibitors or conditional NOS deletion are needed.

### 4.2 S-nitrosylation and myofilaments

Cardiac myofilaments are major targets for SNO, with identified modification sites on both thin filament proteins (actin, troponin complex, tropomyosin) and thick filament proteins (myosin, myosin-binding protein C) (Fig. 2). This PTM serves as a rapid and direct mechanism for fine-tuning cardiac output by modulating myofilament Ca^2+^ sensitivity and cross-bridge cycling kinetics. Although dedicated studies remain limited, with foundational findings derived from *in vitro* models, emerging evidence underscores its physiological and pathophysiological role.

SNO directly modulates the sarcomeric molecular motor. *In vitro* studies demonstrate that nitrosylation of myosin heavy and light chains, using donors like S-nitroso-l-cysteine, reduces actin-sliding velocity and myosin ATPase activity in a dose-dependent manner. Specifically, SNO of myosin light chain 3 (Cys191) prolongs the strongly bound state to actin, increasing the cross-bridge cycle time and thereby altering force generation [143,144].

Under physiological conditions, SNO acts as a dynamic brake on myofilament function, working in concert with canonical pathways like β-adrenergic stimulation to optimize the force-frequency relationship and facilitate relaxation at high heart rates. For example, isoproterenol induces SNO of cardiac TnC at Cys84, reducing myofilament Ca^2+^ sensitivity independently of, and parallel to, TnI phosphorylation [84]. This NO-mediated reduction in Ca^2+^ sensitivity and diastolic stiffness underlies the lusitropic (relaxation-promoting) effect of NO, ensuring efficient ventricular filling and energy conservation. The effect is specific and reversible, as shown in skeletal muscle where SNO of fast TnI (Cys134) decreases Ca^2+^ sensitivity, an effect reversed by reducing agents [145]. Interestingly, SNO and S-glutathionylation exert opposing effects on Ca^2+^ sensitivity in mammalian fast TnI, mediated by competitive actions at Cys134, likely by enhancing TnI's inhibitory action on actomyosin ATPase to promote relaxation.

The thick filament myosin-binding protein C (cMyBP-C) acts as a structural and regulatory brake on contraction. In mouse hearts perfused with the S-nitrosylating agent GSNO, Cys1270 in cMyBP-C was identified as a modification site [146]. SNO of cMyBP-C at this residue is predicted to modulate its interaction with myosin and/or actin, potentially accelerating cross-bridge cycling and contributing to positive lusitropy. However, its *in vivo* functional impact remains unvalidated.

The giant molecular spring protein titin, which determines passive myocardial stiffness, is also a target of SNO. Oxidative stress may induce SNO of sarcomeric proteins, including titin, and thereby depressing myofilament Ca^2+^ sensitivity in intact cardiomyocytes [144]. Although multiple SNO sites have identified in CysNO- exposed cardiomyocytes, whether these modifications affect titin-based passive stiffness remains unclear.

In cardiovascular diseases such as HF and atrial fibrillation, SNO balance is often disrupted, a state known as “nitrosative stress” [118]. Findings from human and experimental HF suggest that a global reduction in SNO occurs during HF with contractile consequences such as increased Ca^2+^ sensitivity due to hypo-nitrosylation of TnI and other targets, impairing relaxation and contributing to diastolic dysfunction. Additionally, reduced titin SNO likely elevates passive tension, further compromising diastolic filling. This pathological increase in stiffness worsens cardiac efficiency and accelerates disease progression. Conversely, ischemic and GSNO preconditioning induces SNO of sarcomeric proteins including myomesin, cardiac alpha-myosin heavy chain, and myosin light chain 1 [142] suggesting a cardioprotective role for SNO.

## 5 S-nitrosylation and cardiac bioenergetic

Mitochondria play a central role in cardiac function by producing ATP, thereby sustaining bioenergetic excitation–contraction coupling. Beyond its role in sarcomere contraction, Ca^2+^ released through RyR2 during ECC is also taken up by mitochondria via the mitochondrial Ca^2+^ uniporter (MCU) complex. The resulting rise in [Ca^2+^]_mt_ stimulates tricarboxylic acid cycle (TCA) dehydrogenase activity, thereby enhancing ATP production. Notably, key regulators of mitochondrial Ca^2+^ homeostasis are sensitive to SNO. For example, the MCU subunit undergoes SNO, which reduces mitochondrial Ca^2+^ uptake and overload [147]. Additionally, TMEM65, a regulator of mitochondrial Na^+^/Ca^2+^ exchange [148], is also S-nitrosylated in the heart [149]. However, the functional and bioenergetic consequences of this redox PTM are unknown. Thus, SNO appears to act as a potential key regulator of mitochondrial Ca^2+^ homeostasis, but further studies are needed to elucidate its impact on myocardial bioenergetics (Fig. 2).

The enhanced TCA activity depends on substrate supply, which in the heart is primarily derived from fatty acid oxidation (FAO, 60–90% of energy) and glycolysis (20–40%) under resting conditions. This metabolic profile can be reprogrammed in response to stress or during pathological conditions. Proteomic studies reveal that proteins involved in metabolic energetic pathways [150], particularly those in FAO [15], are among the most extensively S-nitrosylated. However, few studies have investigated the functional metabolic consequences of this modification. For instance, in a hypertension model, SNO of transglutaminase 2 impairs FAO and cardiomyocyte contraction [151], whereas in the liver, SNO of the very-long-chain acyl-CoA dehydrogenase at Cys238 enhances its activity [15]. Additionally, GAPDH, a glycolytic enzyme involved in transnitrosylation, reduces global mitochondrial protein SNO, including TCA proteins, when deleted [152]. Despite limited data, SNO appears to primarily target proteins regulating cellular energetic processes.

Notably, in a high-fat diet (HFD)-induced type 2 diabetes model, inhibiting NO production with l-NAME exacerbates cardiac metabolic and mitochondrial dysfunctions. In this model, HFD-treated mice with l-NAME develop HFpEF, characterized by severe mitochondrial and energetic metabolic impairments [153]. These findings suggest that NO production plays a key role in protecting the heart against metabolic disorders. Consistently, SNO of TCA proteins is involved in cardioprotection [154,155] and exercise-induced cardioprotection [72]. Suppressing NO signaling either by NOS inhibition (l-NAME) or Cav-3 deletion induces mitochondrial dysfunction and abolishes cardioprotection. By contrast, specific inhibition of guanylate cyclase and PKG does not, highlighting the critical role of SNO [155]. In this work, the SNO of F1-ATPase α-subunit with the NO donor GSNO is associated with a dose-dependent reduction of its activity, which is consistent with the decrease in ATP consumption observed in the ischemic preconditioned hearts [142].

Despite evidence that SNO contributes to cardioprotection via metabolic regulation, in pathological contexts, SNO of proteins involved in cellular energetic processes can exert adverse effects. In a dyssynchronous canine HF model, SNO of ATP synthase subunit at Cys294, induced during cardiac resynchronization therapy, suppresses deleterious oxidation but inhibits ATP synthase activity [156]. In addition, in HF, the SNO of Pyruvate Kinase 2 (Cys49 and 326) decreases its activity and increases mitochondrial fission in mice and human cardiac fibroblasts [157]. The ANT1 is also found to be S-nitrosylated (Cys160) in HF patients, which contributes to cardiac mitochondrial dysfunction [117]. It seems then that SNO of proteins involved in cardiac energy metabolism contributes to the cardioprotective role of NO, however, in some pathological contexts, SNO may exert deleterious effects on cardiac energetics and function.

SNO also regulates mitochondrial quality control [158]. For instance, SNO of the dynamin-related protein 1 (DRP1, Cys644) promotes its phosphorylation at Ser616, enhancing oligomerization [159]. This modification is associated with excessive mitochondrial fragmentation in neurons [159], though no cardiac data currently exist. Similarly, SNO activates parkin [160], but its role in cardiac mitochondrial quality control remains unexplored.

Overall, NO modulates, through SNO, the function of numerous proteins critical to cardiac bioenergetics. While the cardioprotective role of SNO has been relatively well characterized, its metabolic and functional consequences remain incompletely understood and therefore warrant further investigation.

## 6 S-nitrosylation and vascular function

This review aims to highlight the impact of SNO on cardiomyocyte function. However, cardiac contractile performance of these cells depends on metabolic substrate delivery by the vascular system. Therefore, we briefly review below whether this redox post-translational modification (PTM) can also modulate vascular function (Fig. 4).

**Fig. 4:**
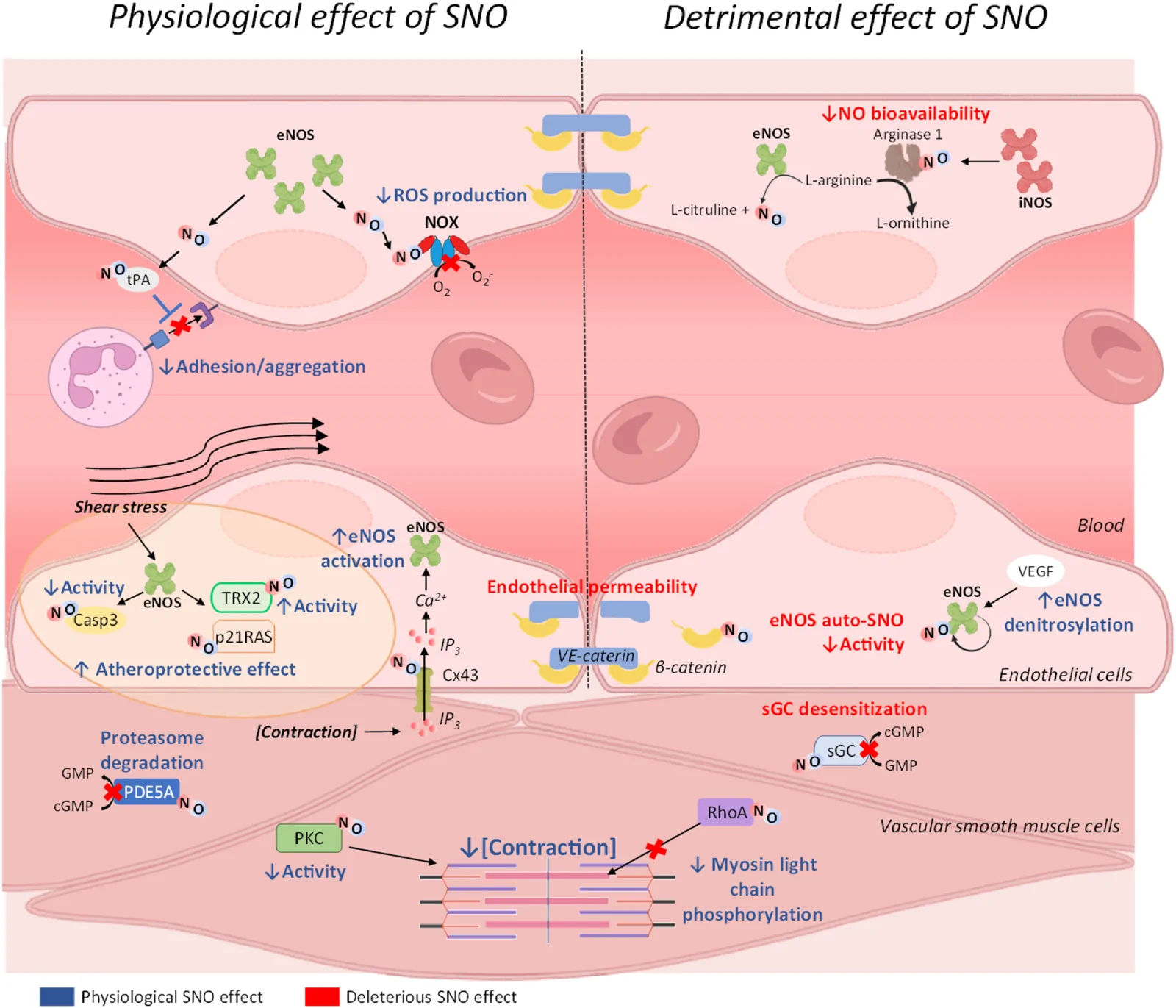
**Functional implication of S-nitrosylation within the vascular function** Beyond its well-established role in vasodilation through the canonical NO-cGMP-PKG pathway, NO also mediates numerous effects on endothelial function through protein S-nitrosylation (SNO). SNO is primarily driven by the activation of endothelial nitric oxide synthase (eNOS), the predominant NOS isoform in endothelial cells, which can be stimulated by both shear stress and vascular smooth muscle cell (VSMC) contraction, thereby increasing NO production. eNOS activation promotes the S-nitrosylation-dependent proteasome degradation of phosphodiesterase 5A (PDE5A), thereby preserving intracellular cGMP levels and maintaining endothelial vasodilatory capacity. In VSMCs, S-nitrosylation of protein kinase C (PKC) and RhoA contributes to the reduction of contractile function. SNO also exerts anti-atherogenic and antioxidant effects by preventing neutrophil adhesion and reducing reactive oxygen species (ROS) production by NADPH oxidases (NOXs). At elevated NO concentrations, however, several feedback mechanisms regulate eNOS activity. In particular, eNOS auto-S-nitrosylation reduces its dimerization and enzymatic activity. Likewise, S-nitrosylation of soluble guanylate cyclase (sGC) reduces cGMP formation and consequently attenuates the vasodilatory response. In addition, iNOS-driven S-nitrosylation of arginase-1 in endothelial cells promotes competition for the common substrate, l-arginine, resulting in endothelial dysfunction. Furthermore, S-nitrosylation of β-catenin disrupts intercellular junctions and increases endothelial permeability.

Numerous studies have demonstrated NO's vasodilatory, anti-atherogenic, and anti-thrombotic effects, initially attributed to activation of the soluble guanylate cyclase–cGMP signaling pathway. However, as previously reviewed [161], NO also modulates vascular homeostasis through direct SNO of regulatory proteins. For example, inhibiting GSNOR, the primary enzyme responsible for protein denitrosylation, induces vasodilation [162], and GSNOR deletion reduces systemic vascular resistance [163]. These effects align with GSNO's role as an endothelium-derived relaxing factor, as both NO and GSNO elevate cGMP levels. Moreover, SNO of phosphodiesterase 5A, the enzyme that hydrolyzes cGMP, promotes its ubiquitin–proteasome–mediated degradation [164]. Consistent with this mechanism, shear stress, a potent eNOS activator, triggers SNO of over 100 proteins [165], including proteins involved in apoptosis (casp-3; reduced activity), cell survival (p21ras) and redox regulation (Trx; increased activity) [166]. These findings suggest that NO's atheroprotective effects are partly mediated by SNO. Supporting this, SNO of tissue plasminogen activator (tPA) inhibits neutrophil–endothelium interactions [167], and NO suppresses NADPH oxidase–derived superoxide production in endothelial cells through SNO of p47phox [168].

Compartmentalized SNO of connexin 43 (Cx43) has emerged as a key regulator of communication between vascular smooth muscle cells (VSMCs) and the endothelium [169]. In an elegant study, Straub et al. demonstrated that S-nitrosylated Cx43 facilitates inositol 1,4,5-trisphosphate (IP_3_) transfer from VSMCs to endothelial cells at myoendothelial junctions, leading to eNOS activation and subsequent NO release in response to smooth muscle contraction. Thus, Cx43 SNO enhances the endothelium's anticontractile function.

SNO also reduces vascular contractility through other targets. For instance, Choi et al. showed that SNO inhibits protein kinase C (PKC)-mediated phosphorylation and activation of contractile proteins in VSMCs [170]. Similarly, Lin et al. demonstrated that S-nitrosothiols attenuate myosin light chain phosphorylation by inhibiting RhoA/Rho-kinase signaling through modification of cysteine residues in the RhoA GTP-binding domain [171]. In addition, S-nitrosylated actin has been proposed to function as a NO donor with rapid vasodilatory activity [172]. More recently, SNO of Ca^2+^-calmodulin-dependent kinase II (CaMKII) [173] and PKC [174] have been identified as critical mechanisms mediating the protective effects of orally administered sodium nitrite against hypertension.

Nonetheless, SNO can also impair vascular function. The endothelial NOS isoform is a central regulator of vascular tone. eNOS undergoes SNO, which reduces its enzymatic activity and impairs dimerization [175], potentially serving as a negative feedback mechanism to limit excessive NO production [176]. Supporting this, vascular endothelial growth factor stimulation induces rapid eNOS denitrosylation (within 5 min) [177], and eNOS subcellular localization influences its SNO status [177].

SNO also desensitizes sGC, the NO receptor. This process, triggered by the NO donor S-nitrosocysteine, is concentration- and time-dependent and may contribute to vascular NO tolerance, thereby inhibiting the eNOS–NO–cGMP pathway and promoting endothelial dysfunction [178]. Additional SNO targets further exacerbate vascular impairment. For example, in age-related endothelial dysfunction, iNOS-dependent SNO increases arginase-1 activity, which competes with eNOS for l-arginine, reducing NO bioavailability [179]. Moreover, SNO of β-catenin increases endothelial permeability, another hallmark of endothelial dysfunction [180].

Overall, these findings demonstrate that SNO's role in vascular function cannot be simplistically categorized as beneficial or detrimental. Rather, its impact depends on the physiological context, cellular compartment, and specific protein targets involved. A more nuanced understanding that integrates spatial localization, NO production levels, and redox balance is essential to fully elucidate the role of SNO in vascular regulation.

## 7 SNO and cardiac response to stress, from physiology to pathology

### 7.1 SNO and response to adrenergic stimulation

β1-and β2-adrenergic receptor (β-AdR) stimulation enhances cardiac chronotropy, inotropy, and lusitropy by phosphorylating key ECC proteins (RyR2, PLB, LTCC, titin, and TnC), thereby boosting contractile capacity. β-AdR activation also increases NO production in cardiomyocytes [181]. This NO surge primarily regulates ECC via the canonical cGMP-PKG pathway [2]. However, the role of protein SNO in cardiac adrenergic response remains less explored (Fig. 5).

**Fig. 5:**
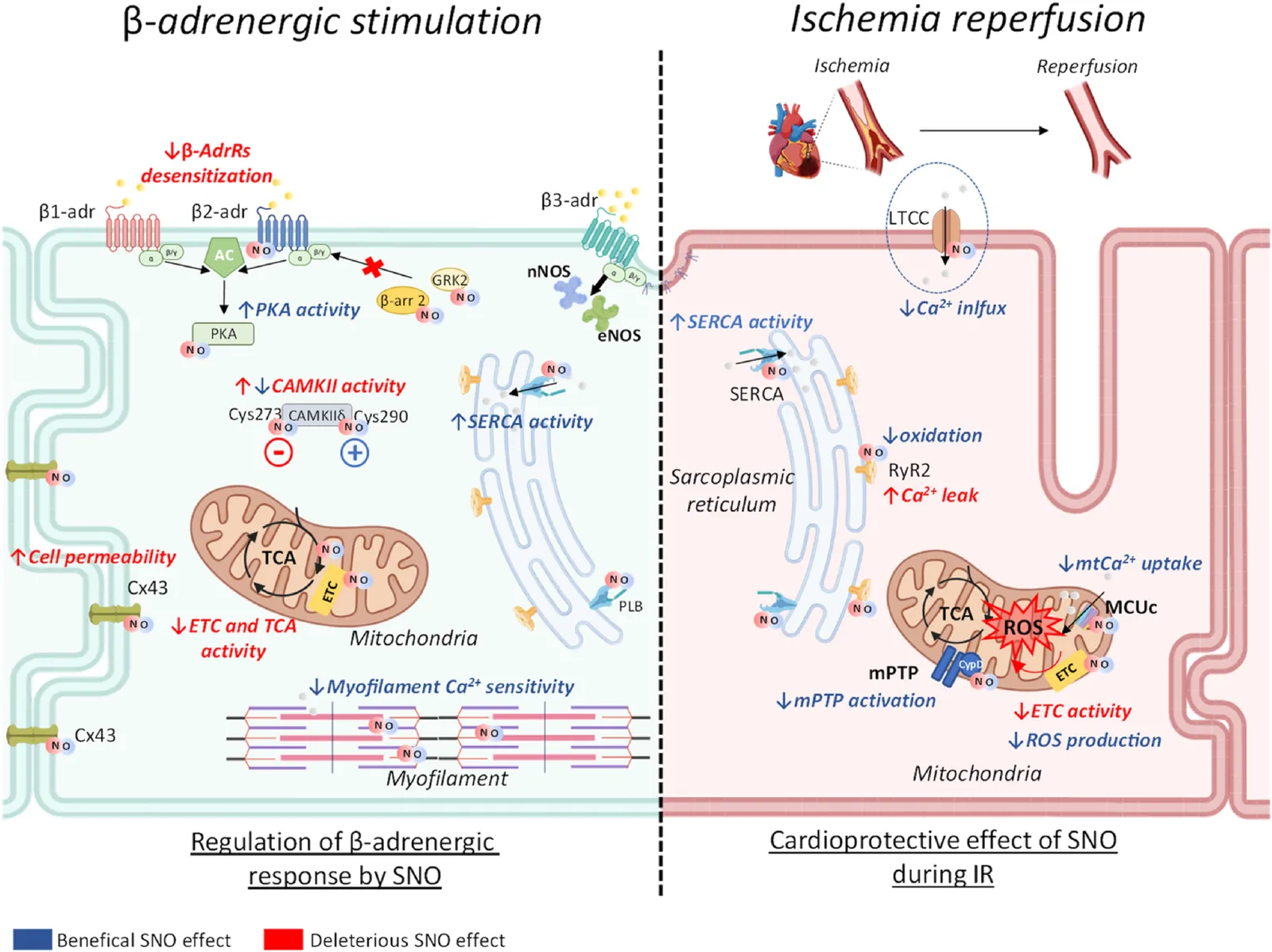
**S-nitrosylation effect during cardiac physiological or pathological stress.** *Left panel*: It is well known that β-adrenergic receptors stimulation is associated with higher NO production. Adrenergic stimulation is well established to promote NO production by activating eNOS and inducing nNOS upregulation. In response to adrenergic stress, S-nitrosylation appears to limit β-adrenergic receptor desensitization and internalization, thereby sustaining β-adrenergic signaling. S-nitrosylation also enhances the positive lusitropic response by potentiating SERCA activity and decreasing myofilament Ca^2+^ sensitivity. In addition, S-nitrosylation may attenuate mitochondrial respiration, potentially limiting reactive oxygen species (ROS) production. Finally, the effects of S-nitrosylation on CaMKII appear to be complex and cysteine residue-dependent, exerting either beneficial effects on the adrenergic response or deleterious effects by promoting arrhythmogenesis. *Right panel:* S-nitrosylation is well established as a mediator of several cardioprotective strategies against ischemia–reperfusion injury, including pharmacological interventions (e.g., MitoSNO), ischemic preconditioning and postconditioning, and exercise. Overall, S-nitrosylation limits cytosolic Ca^2+^ overload by modulating the activity of L-type Ca^2+^ channels (LTCCs), RyR2, and SERCA. At the mitochondrial level, S-nitrosylation is associated with reduced ROS production, decreased Ca^2+^ uptake into the mitochondrial matrix, and inhibition of mitochondrial permeability transition pore (mPTP) opening.

SNO modulates β-AdR signaling at multiple levels. The NO donor S-nitroso-N-acetylcysteine induces β2-AdR SNO, which increases receptor expression and protects against hypertrophy and arrhythmias [182]. During β-adrenergic stimulation, SNO targets G-protein-coupled receptor kinase 2 (GRK2) at Cys340 [26] and β-arrestin 1 and 2 on Cys251 and Cys253, respectively [183]. This reduces β-AdR internalization by inhibiting GRK2-mediated phosphorylation and β-arrestin binding [26]. In HF, iNOS and nNOS upregulation enhance β-arrestin 2 SNO, promoting its dimerization and cytosolic interactions [183]. This mechanism limits β-AdR internalization, thereby delaying cardiac decompensation in a transverse aortic constriction HF model [183]. Consistently, mice expressing SNO-resistant GRK2 (GRK2-C340S) exhibit GRK2 overactivity, reduced β-AdR density, impaired cardiac function, and age-related hypertrophy [184]. These findings demonstrate that SNO preserves physiological β-AdR responsiveness during adrenergic stress.

SNO also fine-tunes canonical β1-and β2-AdR downstream signaling. SNO of PLB at Cys36 and Cys41, and cTnC at Cys84, enhances Ca^2+^ handling during ECC and reduces myofilament Ca^2+^ sensitivity in response to β-adrenergic stimulation [84]. These effects are abolished by the NOS inhibitor l-NAME or in GSNOR-overexpressing mice, confirming that denitrosylation disrupts β-adrenergic Ca^2+^ signaling [84]. RyR2 SNO, as discussed above (6.1.3), involves crosstalk with other PTMs. β-adrenergic stimulation increases RyR2 SNO [129], an effect blocked by the nNOS inhibitor SMTC. However, in GSNOR-deficient mice, impaired β-agonist–induced RyR2 denitrosylation alters the β-adrenergic inotropic response [114]. Thus, rapid, reversible SNO of ECC proteins fine-tunes the cardiac contractile response to β-adrenergic stimulation.

SNO further modulates signaling pathways regulating the cardiac β-adrenergic response. Crosstalk between SNO and kinase phosphorylation has been reported [185]. cAMP-dependent PKA activation is a hallmark of β-adrenergic stimulation; only one study has shown that SNO directly activates type I PKA independently of β-adrenergic stimulation [186]. However, the expected positive inotropic effects are abrogated by simultaneous CysNO-mediated PKGIα activation.

CaMKII modulation by SNO has been more extensively studied [187,188]. The relationship between CaMKII SNO and the cardiac adrenergic response is highly context-dependent. GSNO pretreatment of isolated cardiomyocytes and Langendorff-perfused hearts before isoproterenol stimulation limits Ca^2+^ spark generation and arrhythmias via CaMKIIδ SNO at the inhibitory Cys273 [188]. However, GSNO application after isoproterenol stress causes CaMKIIδ SNO at Cys290, leading to sustained or exacerbated arrhythmias. These findings highlight that the functional impact of CaMKIIδ SNO depends on timing, context, and the specific modification site.

Under chronic β-adrenergic stimulation, SNO dysregulation contributes to maladaptive cardiac remodeling and an altered response to acute β-adrenergic response. In a Duchenne muscular dystrophy mouse model, isoproterenol stress induces aberrant Cx43 activation via SNO at Cys271 [189]. This modification leads to abnormal cell permeability and severe ventricular arrhythmias, which are prevented by inhibiting NO production.

Beyond β1-and β2-AdR isoforms, β3-AdR is expressed in human cardiomyocytes [190]. β3-AdR activation at higher catecholamine concentrations exerts negative inotropic and lusitropic effects, suggesting a protective role during excessive adrenergic stress [191]. This protection primarily involves eNOS and nNOS activation [190]. In eNOS^−/−^ mice, β3-AdR stimulation loses its negative inotropic effect [57] and cardioprotection [192]. nNOS-deficient mice exhibit increased xanthine oxidoreductase-derived ROS and eNOS uncoupling, enhancing the β-adrenergic inotropic response [193]. In the heart, exercise-induced eNOS activation depends on β3-AdR signaling [192]. In this context, increased protein SNO serves as a NO storage form [192,194], which can be released under ischemia through transnitrosylation [192], thereby promoting the SNO of key mitochondrial proteins, including cyclophilin D (CypD), Cx43, NDUFA10 and CHCHD2, that contribute to cardioprotection [72]. These findings underscore the reliance of β3-AdR signaling on NOS enzymes. Furthermore, β3-AdR overexpression or stimulation with the selective agonist BRL exerts cardioprotective effects, reduces fibrosis, and limits hypertrophic remodeling [79,195].

Overall, these findings reveal the multifaceted role of SNO in the cardiac β-adrenergic response. Beyond modulating ECC, SNO regulates mitochondrial function, arrhythmogenic pathways, and β-adrenergic desensitization. Thus, balanced SNO is proposed to be essential for maintaining physiological β-adrenergic responsiveness and preventing maladaptive cardiac remodeling under chronic adrenergic stress.

### 7.2 Ischemia reperfusion

Ischemia/reperfusion (I/R) injury causes myocardial damage and cell death, a major driver of HF [196,197]. While restoring blood flow is essential to salvage ischemic myocardium, early reperfusion paradoxically worsens injury via oxidative stress, Ca^2+^ overload, mitochondrial dysfunction, and mPTP opening, leading to cardiomyocyte necrosis and apoptosis [[198], [199], [200]]. In this context, NO-dependent SNO acts as a critical endogenous cardioprotective mechanism, linking physiological redox regulation to I/R pathology [201,202] (Fig. 5).

SNO's cardioprotection during I/R relies on two complementary principles. First, SNO of specific cysteines prevents irreversible oxidation upon reperfusion, acting as a transient “redox shield” to preserve protein function under oxidative stress [203,204]. Second, SNO is rapidly induced and reversible, allowing transient suppression of metabolism and ROS generation before normal function resumes [205,206]. Supporting these principles, global S-nitrosoproteomic analyses reveal that cardioprotective interventions, including ischemic preconditioning, postconditioning, exercise, and NO donors (MitoSNO, GSNO, and nitrite), all trigger widespread myocardial SNO at reperfusion onset [72,142,155,203,207]. Enhanced early reperfusion SNO correlates with improved contractile [203] and coronary vascular [208] recovery, and reduced infarct size [205].

Mitochondria are the primary hub for NO-dependent cardioprotection, with mitochondrial proteins overrepresented as SNO targets [201]. Proteomics has identified >1000 S-nitrosocysteine sites in nearly 650 proteins, mostly linked to oxidative phosphorylation and intermediary metabolism [209,210]. Under ischemia, mitochondrial SNO is selectively amplified, primarily targeting oxidoreductases and OXPHOS subunits to modulate respiration, ROS production, and mPTP opening [211,212].

A pivotal mechanism is SNO of complex I ND3 subunit (Cys39) during ischemia. MitoSNO, a mitochondria-targeted S-nitrosothiol, induces this modification, driving complex I into a transient low-activity state that reduces ROS production at reperfusion onset, protecting against I/R injury [205]. MitoSNO at reperfusion also reduces infarct size and attenuates post-infarct HF, demonstrating therapeutic potential beyond ischemia [213]. Similar effects are observed with SNO-MPG, which reversibly inhibits complex I activity and cardiomyocyte respiration [214,215]. MitoSNO also reversibly inhibits complex IV by competing with O_2_ at the binuclear center [211,216]. Hou et al. developed a stimuli-responsive mitochondria-targeted NO donor that releases NO in response to elevated mitochondrial superoxide; this compound protected against I/R injury in H9c2 cardiomyocytes and isolated rat hearts [217].

SNO of complex III (cytochrome *bc*1 subunit) and the F1-ATPase α subunit occur in ischemic preconditioning [154], postconditioning [155], and GSNOR inhibition [218], reducing complex activity [142,155]. F1-ATPase α subunit SNO restrains reverse-mode ATP synthase activity, limiting futile ATP hydrolysis during ischemia and preserving cardiomyocyte energy [142,155]. Wang et al. showed that Cys294 of this subunit undergoes SNO, shielding it from irreversible disulfide bond formation [156]; however, this was characterized in cardiac resynchronization therapy, so its relevance to ischemic conditioning requires further study.

SNO of TCA cycle enzymes and creatine kinase in preconditioned/GSNO-treated hearts coordinates a downregulation of mitochondrial electron flux, limiting ROS while preserving ATP [142,155,203]. Another critical target is CypD, the mPTP regulatory subunit. SNO of its Cys203 (mouse)/Cys202 (human) during ischemic preconditioning elevates the Ca^2+^ threshold for pore opening, preventing mitochondrial swelling and cytochrome *c* release [[219], [220], [221]]. The C203S mutation confirms this residue's importance, conferring resistance to Ca^2+^- and H_2_O_2_-induced mPTP opening and reducing infarct size. Exercise training amplifies this protection by upregulating mitochondrial eNOS expression and Ser1177 phosphorylation, enhancing SNO of CypD, Cx43, NDUFA10, and CHCHD2, and improving mitochondrial Ca^2+^ retention capacity [72].

Beyond mitochondria, SNO limits cytosolic Ca^2+^ overload by coordinating Ca^2+^-handling proteins. Ischemic preconditioning-induced SNO of the LTCC α1 subunit reduces sarcolemmal Ca^2+^ influx [204,222]. RyR2 SNO modulates SR Ca^2+^ release [138,222]. GSNO enhances SERCA2a activity during ischemia and early reperfusion, reducing diastolic Ca^2+^ accumulation [142]. Casp-3 SNO at its catalytic site directly suppresses apoptosis [223] [224].However, SNO is not inherently protective; its effects depend on target identity, stoichiometry, and timing. For example, SNO of soluble epoxide hydrolase degrades cardioprotective epoxyeicosatrienoic acids, potentially worsening ischemic injury [225]. Chronic loss of ANT1 denitrosylation (via mitochondrial GSNOR) causes aberrant ANT1 SNO, impairing mitophagy and worsening mitochondrial dysfunction and HF [117]. The narrow therapeutic window for NO donors underscores this: 200 μM GSNO is cardioprotective, but 500 μM fails to reduce infarct size, likely due to excessive nitrosative stress and peroxynitrite formation [226]. This dose-dependent vulnerability is compounded by comorbidities (diabetes, aging, eNOS uncoupling), which amplify nitrosative stress and may explain the limited clinical translation of preconditioning strategies [227,228]. Indeed, elevated nitrosative stress (increased protein SNO and nitrotyrosination) increases I/R vulnerability in obese diabetic mice [229].

Overall, temporally and spatially controlled SNO acts as a reversible post-translational switch, coordinating mitochondrial energetics, ROS production, Ca^2+^ homeostasis, and cell death during I/R injury. The central challenge is selectively enhancing protective SNO while preventing nitrosative stress to translate these insights into clinical cardioprotection [230,231].

### 7.3 Heart failure

HF is a complex syndrome characterized by profound alterations in NO signaling and protein SNO, which display distinct patterns in HF with reduced ejection fraction (HFrEF) and HFpEF, reflecting their divergent underlying pathophysiological mechanisms [118,232] (Fig. 6).

**Fig. 6:**
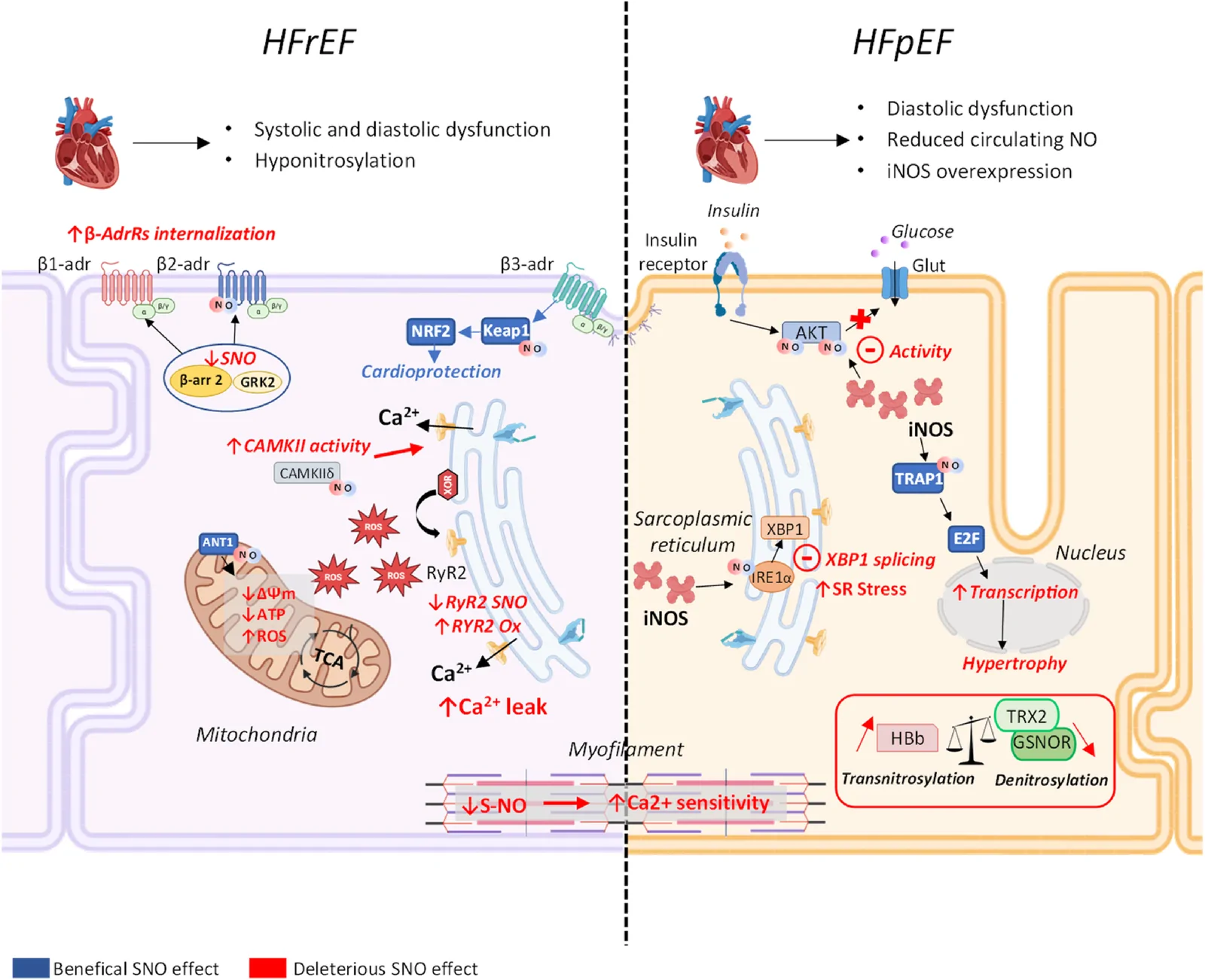
**S-nitrosylation effect during heart failure.** *Left panel*: In HFrEF, reduced S-nitrosylation contributes to several maladaptive mechanisms. First, it promotes β-adrenergic receptor internalization and desensitization, thereby impairing adrenergic responsiveness. Second, it enhances RyR2 oxidation through increased mitochondrial ROS (mtROS) and ROS derived from other sources, leading to pathological Ca^2+^ leak from the sarcoplasmic reticulum. Third, reduced S-nitrosylation increases myofilament Ca^2+^ sensitivity, which may enhance Ca^2+^ buffering by the myofilaments but also impair relaxation, thereby contributing to diastolic dysfunction. Depending on the modified cysteine residue, S-nitrosylation of CaMKII may either support β-adrenergic signaling or exacerbate RyR2-mediated Ca^2+^ leak and arrhythmogenesis. Finally, recent evidence suggests that β3-adrenergic receptor activation promotes Keap1 S-nitrosylation, leading to NRF2 activation and the induction of a cardioprotective transcriptional program. *Right panel:* HFpEF, characterized by diastolic dysfunction in the absence of major systolic impairment, is associated with reduced circulating NO metabolites but marked nitro-oxidative stress within the myocardium. This imbalance is thought to result, at least in part, from iNOS upregulation and reduced activity of the major denitrosylating systems, including GSNOR and TRX2. At the cellular level, excessive S-nitrosylation of Akt exacerbates glucose metabolic dysfunction, while S-nitrosylation of IRE1α impairs XBP1 splicing, thereby promoting endoplasmic reticulum stress. In addition, S-nitrosylation of TRAP1 activates the Rb–E2F transcriptional axis, contributing to cardiomyocyte hypertrophy. Finally, HFpEF is associated with nitro-oxidative modifications of myofilament proteins that increase myofilament Ca^2+^ sensitivity, a key mechanism underlying impaired relaxation and diastolic dysfunction.

In HFrEF, defined by systolic dysfunction, a global hypo-nitrosylation state predominates due to nitroso-redox imbalance [[233], [234], [235], [236]]. The cardiac RyR2 exemplifies this phenomenon: in failing myocardium, its SNO is impaired by xanthine oxidase-derived superoxide production, contributing to diastolic Ca^2+^ leak and depressed contractility [233]. Mechanistically, oxidative stress both depletes NO bioavailability but competes with SNO for the same cysteine residues, converting protective SNO into irreversible oxidative modifications [237]. Notably, xanthine oxidase inhibition restores RyR2 SNO and contractile function, highlighting the therapeutic potential of targeting this pathway in HFrEF [233,238].

Beyond Ca^2+^ handling, SNO critically modulates β-AdR signaling. Under physiological conditions, eNOS-mediated SNO of both GRK2 (Cys340) and β-arrestin 2 restrains receptor desensitization and downregulation, preserving inotropic reserve [183,239,240]. In the pressure-overloaded failing heart, loss of SNO at β-arrestin 2 Cys253 exacerbates β-AdR dysfunction and abolishes the HIF-1α/p53-dependent adaptive angiogenic response, accelerating cardiac deterioration [240]. This hypo-nitrosylation phenotype extends to other Ca^2+^-handling proteins, as nNOS deficiency exacerbates post-myocardial infarction ventricular arrhythmias and mortality through decreased SNO, suggesting that restoring nitroso-redox balance may prevent sudden arrhythmic death [138,241,242]. At the mitochondrial level, denitrosylation of ANT1 protects against HFrEF progression [117]. Indeed, GSNOR deletion aggravates HF via mitochondrial dysfunction and impaired mitophagy. In a mouse model of transverse aortic constriction-induced HF, human β3-AdR overexpression rewires glucose metabolism toward the pentose phosphate pathway, protecting both mitochondrial and cardiac function [243]. These effects involve NOS-dependent SNO of Keap1, enabling NRF2 nuclear translocation, which enhances antioxidant capacity, cardiomyocyte glucose uptake, and mitochondrial function.

Conversely, HFpEF, characterized by diastolic dysfunction with preserved systolic function and driven by systemic comorbidities such as obesity, hypertension, and metabolic syndrome, exhibits a paradoxical profile. At the systemic level, HFpEF (but not HFrEF) is associated with significantly reduced circulating NO metabolites (nitrate and nitrite), indicating a global deficiency in NO production from constitutive NOS isoforms [232]. In contrast, at the myocardial level, iNOS overexpression promotes excessive protein SNO, generating localized nitrosative stress. Schiattarella et al. demonstrated that iNOS-mediated SNO of IRE1α impairs XBP1 splicing, leading to cardiomyocyte dysfunction; this phenotype is rescued by pharmacological or genetic inhibition of iNOS [235]. Similarly, Guo et al. identified Akt as an additional target of iNOS, showing that SNO at Cys224 suppresses Akt activity, impairs insulin-mediated glucose uptake, and induces mitochondrial dysfunction; all of these effects are reversed by iNOS inhibition or knockout [99]. In line with this, iNOS-dependent SNO of TRAP1 activates the Rb–E2F transcriptional axis, thereby promoting transcriptional activity and leading to cardiomyocyte hypertrophy [244]. More recently, Li et al. refined this model in human HFpEF patients and ZSF1 obese rats, showing that disease progression is driven not only by iNOS but by an imbalance between transnitrosylation (hemoglobin-β) and denitrosylation systems (Trx2 and GSNOR), which collectively reshape the pathological nitrosoproteome [118].

In addition, global alterations of myofilament SNO occurs during HF with contractile consequences such as increased Ca^2+^ sensitivity, which can contribute to explain diastolic dysfunction both in HFpEF and HFrEF [118,144].

Collectively, these findings underscore that the directionality of SNO dysregulation—hyponitrosylation in HFrEF *versus* hypernitrosylation in HFpEF —represents a mechanistic bifurcation that fundamentally distinguishes these HF phenotypes, with significant implications for phenotype-specific therapeutic targeting.

## 8 Sex-dependent differences in cardiac S-nitrosylation

Although most studies in the field have historically been conducted in male models, the well-established sex differences in the incidence and manifestation of cardiovascular diseases highlight the importance of considering sex-dependent differences in NO signaling and cardiac SNO [207,245]. These differences have been extensively reviewed by Murphy and Steenbergen [246] and, more recently, by Bers et al. [222]. Rather than revisiting this extensive body of literature, we will focus here on a few key findings and provide additional insights into the mechanisms that may underlie sex-dependent differences in cardiac SNO.

At baseline, female mouse hearts exhibited nearly 65% more SNO protein identifications than male hearts, providing evidence for marked sex-dependent differences in the myocardial SNO proteome [207]. This greater SNO was associated with higher eNOS expression and Ser1177 phosphorylation, together with increased total NO production in female hearts. Importantly, similar sex-dependent differences have also been reported in non-failing human hearts, although with distinct patterns of subcellular localization: proteins preferentially S-nitrosylated in women were predominantly mitochondrial, whereas those preferentially S-nitrosylated in men were mainly cytosolic [247]. Collectively, these findings suggest that sex differences in cardiac SNO extend beyond the overall abundance of SNO-modified proteins and may also involve distinct subcellular targets. Interestingly, NO production is known to fluctuate across the menstrual cycle, as initially demonstrated by changes in circulating plasma NO levels [248]. Subsequent studies have shown that sex hormones, particularly estrogen, can modulate NOS expression and activity [222,249]. As extensively discussed by Murphy and Steenbergen [246], estrogen-dependent activation of NO signaling contributes to sex differences in cardiac responses, with important consequences for mitochondrial function. At the cardiomyocyte level, estrogen receptor-β (ERβ) activation promotes eNOS activation through the ERβ/PI3K/Akt pathway, thereby increasing SNO of mitochondrial proteins, including CypD and components of the electron transport chain [222,250]. Thus, estrogen-dependent modulation of eNOS activity may represent an important mechanism linking sex hormones to mitochondrial S-nitrosylation and cardiac stress responses.

These sex-dependent differences under basal conditions may contribute to distinct cardiac response to stress. Indeed, in the context of I/R, female hearts display greater resilience to injury, with significantly better functional recovery than male hearts [207]. This cardioprotection has been linked, at least in part, to SNO of the LTCC α1C subunit by eNOS and nNOS, which slows cellular Ca^2+^ entry and limits reperfusion injury [251]. More recently, sex-dependent differences have also been identified at the mitochondrial level, with female hearts exhibiting reduced mitochondrial Ca^2+^ overload and decreased sensitivity to mPTP opening during reperfusion [147]. During ischemia, female hearts likewise display a smaller rise in mitochondrial Ca^2+^, thereby limiting Ca^2+^ accumulation and preserving mitochondrial bioenergetics. Notably, this protection was associated with SNO of the MCU, representing a novel mechanism by which SNO attenuates MCU activity and limits mitochondrial Ca^2+^ accumulation [147]. Collectively, these findings suggest that sex-dependent differences in SNO may contribute to cardioprotection by modulating Ca^2+^ handling during ischemic stress.

Conversely, sex-dependent SNO of SERCA2a at Cys344 has been reported to be more than threefold higher in female than in male hearts [207] and is associated with reduced isoproterenol-induced SR Ca^2+^ loading [252]. Notably, this sex difference was abolished by NOS inhibition, supporting a role for NO-dependent signaling in the differential regulation of SERCA2a activity between female and male hearts [252].

Although differences in NOS expression and/or activity may contribute to the sex-dependent regulation of SNO-mediated cardioprotection, they are unlikely to fully account for these differences. Other key determinants of SNO homeostasis, particularly the major denitrosylating enzyme GSNOR, may also contribute to the distinct SNO profiles observed between female and male hearts. Indeed, GSNOR inhibition or deletion is cardioprotective in male hearts but exerts opposite effects in female hearts [116]. This sex-dependent difference has been proposed to involve differential SNO of the mitochondrial complex I ND3 subunit in male and female hearts. Indeed, pharmacological inhibition of GSNOR increased ND3 SNO in male hearts, whereas an opposite effect was observed in female hearts. Consistent with these distinct responses, GSNOR inhibition reduced mitochondrial ROS production following I/R in male hearts, but not in female hearts. These findings suggest that GSNOR may play a sex-specific role in regulating mitochondrial SNO and ROS production and may be particularly important for maintaining cardioprotection in the female heart during I/R [116]. Interestingly, GSNOR expression and activity have been shown to decline with age in female hearts [253]. Given the proposed role of GSNOR in maintaining sex-dependent SNO homeostasis and cardioprotection, this age-related decline may contribute to the reduced cardioprotective capacity of the female heart with aging.

While healthy female hearts appear to exhibit a greater degree of SNO than male hearts, this pattern is reversed in the setting of heart failure. Indeed, the reduction in protein SNO appears to be more pronounced in failing female hearts, with 71% of detected proteins showing decreased SNO compared with 43% in failing male hearts [247]. In female failing hearts, these alterations were associated with increased eNOS S-glutathionylation, enhanced eNOS uncoupling, and disruption of nitroso-redox signaling, suggesting a shift from a more S-nitrosylated basal state toward impaired NO/SNO signaling during cardiac disease.

Altogether, these findings suggest that, under basal conditions, NO signaling in the female heart represents an important determinant of cardioprotection, notably through the regulation of Ca^2+^ homeostasis and mitochondrial function. However, the greater reliance on NO/SNO signaling in the female heart may also have important consequences under pathological conditions, such that disruption of this pathway could result in a more pronounced loss of cardioprotection in females. Alterations in NO signaling, and particularly in protein SNO, may therefore contribute to the emergence of sex-dependent differences in cardiovascular disease susceptibility and progression. Nevertheless, the mechanisms underlying these sex-dependent effects remain incompletely understood and warrant further investigation.

## 9 Therapeutic implications and clinical translation

Protein SNO offers a novel therapeutic target for cardiovascular diseases, with phenotype-specific strategies required for optimal efficacy.

Enhancing Protective SNO: In HFrEF and I/R injury, restoring SNO of key cardioprotective targets offers a promising approach. Mitochondria-targeted S-nitrosothiols (e.g., MitoSNO, SNO-MPG) selectively S-nitrosylate Complex I (ND3 Cys39), transiently inhibiting its activity to reduce ROS production at reperfusion, thereby decreasing infarct size in mouse models [205,[213], [214], [215]]. Similarly, GSNOR inhibition (N6022) globally enhances SNO, preserving RyR2 function by preventing oxidative modification, reducing SR Ca^2+^ leak, and suppressing arrhythmias in nNOS^−/−^ mice [115,138,139]. β3-AdR agonists (BRL-37344, CL-316243) stimulate eNOS-derived NO, increasing mitochondrial SNO of CypD (Cys203) and NDUFA10, which elevates the mPTP opening threshold and protects against I/R injury [73,192]. Additionally, SERCA2a SNO via NO donors (GSNO) or ischemic preconditioning enhances Ca^2+^ reuptake, improving lusitropy in cardiomyocytes [143]. This approach is supported by the “redox shield” hypothesis, where SNO of critical cysteines transiently protects against irreversible oxidation during stress. However, these approaches face challenges: MitoSNO's therapeutic window is narrow (200 μM protective vs. 500 μM harmful) [226], GSNOR inhibition shows sex-specific effects (protective in males but not females) [116], and systemic NO donors lack cardiac specificity.

Inhibiting Pathological SNO: In HFpEF, where nitrosative stress drives pathology, targeting excessive SNO is critical. iNOS inhibition (1400W, L-NIL) or genetic deletion reduces SNO of Akt (Cys224), IRE1α, and TRAP1, restoring insulin signaling, XBP1 splicing, and preventing cardiomyocyte hypertrophy in mouse models [99,235,244]. Xanthine oxidase inhibitors (allopurinol, oxypurinol) reduce superoxide production, preserving NO bioavailability and enhancing RyR2 SNO, thereby improving Ca^2+^ handling and symptoms in HF patients [233,238]. Inhibiting hemoglobin-β-mediated transnitrosylation or TRAP1 SNO disrupts the Rb–E2F axis, mitigating nitrosative stress and hypertrophy in ZSF1 obese rats [118,244]. Yet, iNOS inhibition risks immunosuppression [97], and broad denitrosylation strategies may disrupt physiological SNO signaling.

**Clinical Outlook**: The key to successful translation lies in compartment-specific modulation: mitochondria-targeted NO donors for HFrEF/I/R injury and iNOS inhibitors for HFpEF. Future therapies must address the narrow therapeutic window, sex differences, and comorbidity dependence, potentially through controlled-release formulations, selective denitrosylase inhibitors, or combination approaches (e.g., NO donors + antioxidants). With these advances, SNO-based therapies could revolutionize the treatment of heart failure and I/R injury.

## 10 Conclusion

Protein SNO, though historically understudied due to its labile and redox-dependent nature, emerges as a central regulator of cardiac physiology, modulating excitation–contraction coupling, cellular energetics, and adaptive responses to stress. While SNO provides critical cardioprotection during acute challenges, its dysregulation actively contributes to pathological remodeling, potentially serving as a common mechanistic denominator in the development and progression of heart failure phenotypes. These insights not only advance our understanding of cardiac pathophysiology but also establish SNO signaling as a compelling therapeutic target for future cardiovascular interventions.

## CRediT authorship contribution statement

**Maxime Gouin-Gravezat:** Writing – review & editing, Writing – original draft, Conceptualization. **Doria Boulghobra:** Writing – review & editing, Writing – original draft, Conceptualization. **Antoine Grandperrin:** Writing – review & editing. **Beatrice Alpha-Bazin:** Writing – review & editing, Writing – original draft. **Olivier Cazorla:** Writing – review & editing, Writing – original draft. **Cyril Reboul:** Writing – review & editing, Writing – original draft, Supervision, Conceptualization.

## Funding

This work was supported by a French National Research Agency by Agence Nationale de la Recherche. Replace Fédération Française de Cardiologie by : This work was supported by a grant from the Fédération Française de Cardiologie (NO-Limit Project).

## Declaration of competing interest

The authors have no conflict of interest.

## Data Availability

No data was used for the research described in the article.
